# Supplementary material for: FUSED-Net: Detecting traffic signs with limited data
Source: PLoS One. 2026 Oct 7;21(10):e0359293. doi: 10.1371/journal.pone.0359293 (PMC13645229; doi:10.1371/journal.pone.0359293)
Supplement: Peer review history

# Peer review: Decision Letter 0

**DOI:** 10.1371/journal.pone.0359293.r001

**Authors:** Baicang Guo

23 Jul 2026

Dear Dr. Kabir,

Thank you for submitting your manuscript to PLOS ONE. After careful consideration, we feel that it has merit but does not fully meet PLOS ONE’s publication criteria as it currently stands. Therefore, we invite you to submit a revised version of the manuscript that addresses the points raised during the review process.

Please submit your revised manuscript by Sep 21 2026 11:59PM. If you will need more time than this to complete your revisions, please reply to this message or contact the journal office at plosone@plos.org. When you're ready to submit your revision, log on to https://www.editorialmanager.com/pone/ and select the 'Submissions Needing Revision' folder to locate your manuscript file.

- A letter that responds to each point raised by the academic editor and reviewer(s). You should upload this letter as a separate file labeled 'Response to Reviewers'.
- A marked-up copy of your manuscript that highlights changes made to the original version. You should upload this as a separate file labeled 'Revised Manuscript with Track Changes'.
- An unmarked version of your revised paper without tracked changes. You should upload this as a separate file labeled 'Manuscript'.

If you would like to make changes to your financial disclosure, please include your updated statement in your cover letter. Guidelines for resubmitting your figure files are available below the reviewer comments at the end of this letter.

If applicable, we recommend that you deposit your laboratory protocols in protocols.io to enhance the reproducibility of your results. Protocols.io assigns your protocol its own identifier (DOI) so that it can be cited independently in the future. For instructions see: https://journals.plos.org/plosone/s/submission-guidelines#loc-laboratory-protocols. Additionally, PLOS ONE offers an option for publishing peer-reviewed Lab Protocol articles, which describe protocols hosted on protocols.io. Read more information on sharing protocols at https://plos.org/protocols?utm_medium=editorial-email&utm_source=authorletters&utm_campaign=protocols.

As the corresponding author, your ORCID iD is verified in the submission system and will appear in the published article. PLOS supports the use of ORCID, and we encourage all coauthors to register for an ORCID iD and use it as well. Please encourage your coauthors to verify their ORCID iD within the submission system before final acceptance, as unverified ORCID iDs will not appear in the published article. *Only*  the individual author can complete the verification step; PLOS staff *cannot*  verify ORCID iDs on behalf of authors.

We look forward to receiving your revised manuscript.

Kind regards,

Baicang Guo

Academic Editor

PLOS One

Journal Requirements:

When submitting your revision, we need you to address these additional requirements.

1. Please ensure that your manuscript meets PLOS ONE's style requirements, including those for file naming. The PLOS ONE style templates can be found at

https://journals.plos.org/plosone/s/file?id=wjVg/PLOSOne_formatting_sample_main_body.pdf and

https://journals.plos.org/plosone/s/file?id=ba62/PLOSOne_formatting_sample_title_authors_affiliations.pdf

2. Thank you for stating in your Funding Statement: [This work was supported in part by the Islamic University of Technology (IUT) Seed Grant 2022 (Grant No. REASP/IUT-RSG/2022/OL/07/011) awarded to Dr. Md. Hasanul Kabir. The funder’s website is https://www.iutoic-dhaka.edu/. The sponsor had no role in the study design, data collection and analysis, decision to publish, or preparation of the manuscript].

Please provide an amended statement that declares *all* the funding or sources of support (whether external or internal to your organization) received during this study, as detailed online in our guide for authors at http://journals.plos.org/plosone/s/submit-now.  Please also include the statement “There was no additional external funding received for this study.” in your updated Funding Statement.

Please include your amended Funding Statement within your cover letter. We will change the online submission form on your behalf.

3. PLOS requires an ORCID iD for the corresponding author in Editorial Manager on papers submitted after December 6th, 2016. Please ensure that you have an ORCID iD and that it is validated in Editorial Manager. To do this, go to ‘Update my Information’ (in the upper left-hand corner of the main menu), and click on the Fetch/Validate link next to the ORCID field. This will take you to the ORCID site and allow you to create a new iD or authenticate a pre-existing iD in Editorial Manager.

4. We note that Figures 2,3,5,7 and 8 in your submission contain copyrighted images. All PLOS content is published under the Creative Commons Attribution License (CC BY 4.0), which means that the manuscript, images, and Supporting Information files will be freely available online, and any third party is permitted to access, download, copy, distribute, and use these materials in any way, even commercially, with proper attribution. For more information, see our copyright guidelines: http://journals.plos.org/plosone/s/licenses-and-copyright.

We require you to either (1) present written permission from the copyright holder to publish these figures specifically under the CC BY 4.0 license, or (2) remove the figures from your submission:

1. You may seek permission from the original copyright holder of Figures 2,3,5,7 and 8 to publish the content specifically under the CC BY 4.0 license.

We recommend that you contact the original copyright holder with the Content Permission Form (http://journals.plos.org/plosone/s/file?id=7c09/content-permission-form.pdf) and the following text:

“I request permission for the open-access journal PLOS ONE to publish XXX under the Creative Commons Attribution License (CCAL) CC BY 4.0 (http://creativecommons.org/licenses/by/4.0/). Please be aware that this license allows unrestricted use and distribution, even commercially, by third parties. Please reply and provide explicit written permission to publish XXX under a CC BY license and complete the attached form.”

Please upload the completed Content Permission Form or other proof of granted permissions as an ""Other"" file with your submission.

In the figure caption of the copyrighted figure, please include the following text: “Reprinted from [ref] under a CC BY license, with permission from [name of publisher], original copyright [original copyright year].”

2. If you are unable to obtain permission from the original copyright holder to publish these figures under the CC BY 4.0 license or if the copyright holder’s requirements are incompatible with the CC BY 4.0 license, please either i) remove the figure or ii) supply a replacement figure that complies with the CC BY 4.0 license. Please check copyright information on all replacement figures and update the figure caption with source information. If applicable, please specify in the figure caption text when a figure is similar but not identical to the original image and is therefore for illustrative purposes only.

5. If the reviewer comments include a recommendation to cite specific previously published works, please review and evaluate these publications to determine whether they are relevant and should be cited. There is no requirement to cite these works unless the editor has indicated otherwise.

Additional Editor Comments:

The manuscript presents extensive quantitative evaluations, but an important weakness is the lack of sufficient qualitative visualization results. For an object detection study, representative detection examples are essential to demonstrate the practical effectiveness, robustness, and failure modes of the proposed method. At present, the manuscript relies predominantly on numerical comparisons, while visual evidence supporting the claimed performance improvements is very limited. Consequently, it is difficult to assess whether the proposed approach consistently produces better detection results in realistic scenarios or whether the reported improvements are primarily reflected in benchmark metrics.

Given that qualitative evaluation is a fundamental component of object detection research, I consider the current experimental evidence incomplete. The manuscript should provide substantially more representative detection results to support its claims. Without such evidence, the conclusions regarding the superiority and robustness of the proposed method are insufficiently supported, and I do not believe the manuscript is suitable for publication in its current form.

**With respect to the references suggested during peer review, only those that are strictly relevant to the scope and technical content of the manuscript should be incorporated. Citations should be included based on scientific relevance rather than solely to accommodate reviewer requests.**

[Note: HTML markup is below. Please do not edit.]

Reviewers' comments:

Reviewer's Responses to Questions

**Comments to the Author**

1. Is the manuscript technically sound, and do the data support the conclusions?

Reviewer #1: Yes

Reviewer #2: Yes

**********

2. Has the statistical analysis been performed appropriately and rigorously?

Reviewer #1: Yes

Reviewer #2: No

**********

3. Have the authors made all data underlying the findings in their manuscript fully available?

The PLOS Data policy

Reviewer #1: Yes

Reviewer #2: Yes

**********

4. Is the manuscript presented in an intelligible fashion and written in standard English?

Reviewer #1: Yes

Reviewer #2: Yes

**********

Reviewer #1: 1. The paper does not clearly articulate what is fundamentally new beyond combining these existing techniques. The term "FUSED-Net" appears to be a re-packaging of known methods rather than a conceptually novel framework.

2. The choice of only Color Jitter over other augmentations is not rigorously justified. The claim that it "closely mimics real-world variations" is subjective and unsupported by quantitative analysis.

3. The number of trials for statistical significance is mentioned but not clearly reported (e.g., standard deviations, confidence intervals in tables). The computational cost (e.g., FLOPs, inference time, GPU memory) is not reported, making it difficult to assess practical viability.

4. Some most related works should be added in this paper:

[1]Geometry-Guided Point Generation for 3D Object Detection, IEEE Signal Processing Letters, 2025. DOI: 10.1109/LSP.2024.3503359.

[2]ASCFormer: An Adaptive Strucure-aware Cascaded Transformer for 3D Object Detection. IEEE Transactions on Circuits and Systems for Video Technology, 2026. DOI: 10.1109/TCSVT.2025.3612592.

[3]Soft-Label Guided Multi-Granularity Prompts Learning for Human-Object Interaction Detection, Applied Soft Computing. DOI: 10.1016/j.asoc.2026.114765.

Reviewer #2: This paper introduces FUSED-Net, a novel approach for Few-Shot Object Detection (FSOD) specifically tailored for traffic sign detection in data-scarce environments. The authors address the impracticality of curating large-scale annotated datasets by integrating Faster R-CNN with four key components: unfrozen parameters, pseudo-support sets, embedding normalization, and domain adaptation. However, it remains many problems.

1. The paper states that Color Jitter alone yielded the highest mAP scores after comparing it with other augmentations. However, the details of these comparative experiments are not provided.

2. Include a table or list of key hyperparameters used for training (e.g., learning rate schedule, optimizer settings, number of epochs/iterations) for both pre-training and fine-tuning.

3. Ensure all figures are high-resolution and clearly convey the intended message. For Figure 8, add a more descriptive caption explaining the failure mode.

4. Regarding the "embedding normalization" using a cosine similarity-based classifier, how does the scaling factor γ (set to 20) specifically influence the separation of classes and the reduction of intra-class variance in the context of traffic signs?

5. More related works should be added in revision: https://doi.org/10.1007/s11432-024-4880-x, https://doi.org/10.3390/electronics14102044, and https://arxiv.org/abs/2602.13293v2.

**********

what does this mean?). If published, this will include your full peer review and any attached files.

If you choose “no”, your identity will remain anonymous but your review may still be made public.

**Do you want your identity to be public for this peer review?**  For information about this choice, including consent withdrawal, please see our Privacy Policy

Reviewer #1: No

Reviewer #2: No

**********

[NOTE: If reviewer comments were submitted as an attachment file, they will be attached to this email and accessible via the submission site. Please log into your account, locate the manuscript record, and check for the action link "View Attachments". If this link does not appear, there are no attachment files.]

To ensure your figures meet our technical requirements, please review our figure guidelines: https://journals.plos.org/plosone/s/figures

You may also use PLOS’s free figure tool, NAAS, to help you prepare publication quality figures: https://journals.plos.org/plosone/s/figures#loc-tools-for-figure-preparation.

NAAS will assess whether your figures meet our technical requirements by comparing each figure against our figure specifications.

# Author comment: Author response to Decision Letter 1

**DOI:** 10.1371/journal.pone.0359293.r002

12 Aug 2026

Thank you for the review of our manuscript and for the opportunity to submit a revision. We are very grateful for the encouraging feedback and the insightful suggestions for improvement. We have carefully considered all the comments from the editor and the reviewers and have revised the manuscript accordingly. We have structured our response below to address the provided feedback, and we believe the manuscript is now significantly stronger as a result. We would like to request the editors and reviewers to check the Response to Reviewers PDF (r1-response-july2026.pdf) for better formatting and structure. However, the responses are also provided here for convenience:

Editor Concern 1:

We prepared the manuscript using the LaTeX template (https://journals.plos.org/plosone/s/latex) provided by PLOS ONE. Following the editor's comments, we carefully reviewed the manuscript once again to ensure compliance with the journal's formatting and style requirements. To the best of our knowledge, the manuscript now fully adheres to the formatting guidelines specified by PLOS ONE. In addition, we have updated the filenames of the attached image files to conform to the journal's file-naming requirements.

Editor Concern 2:

We sincerely thank the Editor for pointing out the need to provide a complete declaration of all funding and sources of support received during the study. We have carefully considered this request and have amended the Funding Statement accordingly.

We confirm that the Islamic University of Technology (IUT) Seed Grant 2022 (Grant No. REASP/IUT-RSG/2022/OL/07/011), awarded to Dr. Md. Hasanul Kabir, is the only funding or source of financial support received for this study. No other external or internal funding was received.

The amended Funding Statement is:

"This work was supported in part by the Islamic University of Technology (IUT) Seed Grant 2022 (Grant No. REASP/IUT-RSG/2022/OL/07/011), awarded to Dr. Md. Hasanul Kabir. The funder’s website is https://www.iutoic-dhaka.edu/. The sponsor had no role in the study design, data collection and analysis, decision to publish, or preparation of the manuscript. There was no additional external funding received for this study."

As requested, we have also included this amended Funding Statement in the revised cover letter. We appreciate the Editor's guidance in ensuring that the funding information is complete and transparent.

Editor Concern 3:

Thank you for your comment. We have updated the corresponding author's profile in Editorial Manager to include and validate their ORCID iD, as requested. We appreciate your guidance and assistance in this regard.

Editor Concern 4:

Thank you for bringing this copyright and licensing issue to our attention. We carefully reviewed the licensing conditions of all datasets used in our manuscript and contacted the authors/copyright holders of the relevant datasets to seek permission for publication under the CC BY 4.0 license.

For the Merged Traffic Sign Detection Dataset (MTSD), the samples were drawn from the German Traffic Sign Detection Benchmark (GTSDB), the LISA Traffic Sign Dataset, and the DFG Traffic Sign Dataset. We found that these datasets have licensing conditions that are not directly compatible with the CC BY 4.0 licensing requirements of PLOS ONE: GTSDB is distributed under the CC BY-NC 4.0 license, the LISA Traffic Sign Dataset is released under an academic license agreement, and the DFG Traffic Sign Dataset is distributed under the CC BY-NC-SA 4.0 license, with commercial use requiring additional permission.

We contacted the authors/copyright holders of these datasets to request permission to publish the relevant images specifically under the CC BY 4.0 license. However, we have not received the necessary permissions at this time. Therefore, in accordance with the editorial instructions, we have modified Figure 2 to remove the copyrighted images and removed the figure containing samples from these datasets (previously Figure 3) from the manuscrip. We have also revised the corresponding discussion in the manuscript so that it no longer relies on or refers to the removed figure.

Regarding the Bangladeshi Traffic Sign Dataset (BDTSD), we contacted the corresponding author and received permission to use the dataset/images in our work. We will retain the figures utilizing BDTSD images and have added an appropriate permission statement to the captions of these figures. A copy of the written permission from the corresponding author will also be provided as an ``Other'' file with the revised submission.

We appreciate the editor's guidance, which has helped us ensure that the revised manuscript complies with PLOS ONE's copyright and licensing requirements.

Editor Concern 5:

We sincerely thank the Editor for this clarification. We carefully reviewed and evaluated all publications recommended by the reviewers and assessed their relevance to the scope and contributions of our manuscript. Based on this evaluation, we have incorporated the recommended works that provide relevant context to our discussion of recent advances in object detection and the robustness and reliability of traffic sign perception systems. The corresponding citations have been added at appropriate locations in the Introduction section of the revised manuscript.

Reviewer 1, Concern 1:

We thank the reviewer for this valuable observation. We agree that FUSED-Net is not proposed as an entirely new object detection architecture. Rather, our contribution lies in the design of a unified framework that systematically addresses the unique challenges of few-shot traffic sign detection by integrating several complementary techniques into the standard Faster R-CNN framework. While some of the individual components have been explored in other domains, their adaptation, integration, and validation for cross-domain few-shot traffic sign detection constitute the primary novelty of our work.

Specifically, the novelty of FUSED-Net can be summarized as follows:

- Fully unfrozen fine-tuning: Unlike conventional few-shot object detection approaches that freeze a portion of the network during adaptation, FUSED-Net fine-tunes the entire detector, including the backbone, FPN, RPN, and ROI heads. This enables the model to learn domain-specific representations that are crucial for adapting from general object detection datasets to region-specific traffic sign datasets.

- Pseudo Support Set (PSS): We introduce a Pseudo Support Set generated through targeted color-based augmentation of the limited support samples. To the best of our knowledge, this is the first work to explicitly employ a pseudo support set augmentation strategy for few-shot object detection. The proposed PSS increases the diversity of the support set and improves robustness against illumination, weather, and appearance variations that are frequently encountered in real-world traffic sign images.

- Embedding Normalization (EN): We incorporate a cosine similarity-based classifier with embedding normalization into the traffic sign detection framework. Although cosine similarity classifiers have been investigated in prior few-shot object detection methods, their application to cross-domain few-shot traffic sign detection has not been explored. We demonstrate that embedding normalization effectively reduces intra-class variation and improves feature discrimination among visually similar traffic sign categories.

- Domain adaptation: FUSED-Net is explicitly designed for cross-domain traffic sign detection by leveraging traffic sign datasets collected from multiple countries. This reduces the distribution gap between the source and target domains, resulting in a detector that generalizes more effectively when only a few labeled target-domain samples are available.

Therefore, the contribution of FUSED-Net extends beyond combining existing techniques. Instead, it presents a carefully designed framework in which these complementary components are adapted and integrated to address the specific challenges of few-shot traffic sign detection. Furthermore, the ablation studies presented in the manuscript demonstrate that each proposed component contributes independently to the overall performance, while their combination yields state-of-the-art detection accuracy (62\% improvement over second best method) on the Bangladeshi traffic sign detection benchmark.

Reviewer 1, Concern 2:

We thank the reviewer for raising this important concern. Initially, our intuition was that employing multiple augmentation techniques on the limited samples available in few-shot scenarios could lead to over-complication, potentially impairing the model's ability to learn. Guided by this intuition and prior literature [1], we focused on using Color Jitter, which has been shown to enhance model performance in tasks requiring visual representations. Upon empirical analysis, [1] specifically highlighted that strong Color Jitter achieves superior results, and we adopted their recommended probability of $0.8$ in our implementation.

To further address the comment, we conducted a set of experiments comparing the effectiveness of different popular augmentation strategies, including random cropping, horizontal flipping, and combinations thereof, alongside Color Jitter. The results shown in Table 1 demonstrate that Color Jitter alone outperforms other augmentation strategies and their combinations, achieving the highest average mAP across all shot scenarios. These results validate our decision to use Color Jitter exclusively, and reinforce its effectiveness in introducing realistic variations than other techniques into the support set, making it well suited for our target domain, where traffic signs may appear under diverse real-world conditions, including varying illumination (daytime vs. nighttime) and weather conditions (strong shadows vs. foggy environments). Thus, Color Jittering provides an effective and targeted augmentation strategy for the PSS.

Table 1: Comparison of mAP Performance of FUSED-Net with different augmentation techniques on the Bangladeshi Traffic Sign Detection (BDTSD) Dataset under 1-shot, 3-shot, 5-shot, and 10-shot scenarios.

Color Jitter | Crop | Horizontal Flip | 1-shot | 3-shot | 5-shot | 10-shot | Mean

-------------|------|-----------------|--------|--------|--------|---------|------

✓ | ✗ | ✗ | 28.22 | 44.49 | 47.23 | 54.95 | 43.72

✗ | ✓ | ✗ | 25.00 | 41.42 | 46.35 | 54.46 | 41.81

✗ | ✗ | ✓ | 23.03 | 36.02 | 42.92 | 54.97 | 39.23

✓ | ✓ | ✗ | 26.53 | 42.42 | 46.70 | 51.18 | 41.71

✓ | ✗ | ✓ | 21.53 | 35.75 | 42.81 | 52.87 | 38.29

✗ | ✓ | ✓ | 21.27 | 36.85 | 42.00 | 55.37 | 38.87

✓ | ✓ | ✓ | 23.42 | 40.05 | 44.78 | 50.43 | 39.67

To address the reviewer's concern, we have included Table 1 in the Ablation Study, along with the corresponding analysis on Page 18 (Line 671 onwards). We have also clarified how Color Jittering is applied in the Implementation Details at Page 12 (line 463 onwards).

Reviewer 1, Concern 3:

We thank the reviewer for pointing out the need for clearer reporting of statistical significance and computational cost. We agree that these analyses strengthen the scientific rigor of the study and demonstrate the practical viability of FUSED-Net.

To address the statistical significance concern, we conducted seven independent trials of FUSED-Net and report the mean mAP with 95\% confidence intervals (CI95) for the 1-, 3-, 5-, and 10-shot settings in Table 2. The consistently narrow confidence intervals demonstrate stable performance across runs. We have also updated Figure 5 in the revised manuscript to show the quantitative variation for each bar and revised the Statistical Significance of the Performance subsection accordingly in Page 15 (Line 579 onwards).

Table 2: Mean mAP of FUSED-Net across different shot scenarios with 95% confidence intervals.

Setup | Mean | CI95

-------|-------|------

1-shot | 26.64 | ± 1.27

3-shot | 44.22 | ± 1.15

5-shot | 45.94 | ± 0.45

10-shot| 54.44 | ± 0.75

To address practical viability, we additionally report GFLOPs, average inference time (seconds/sample), and peak GPU memory (GB) for all compared methods in Table 3, measured using an RTX 2000 Ada with 16 GB VRAM. FUSED-Net, TFA w/cos, and FRCN-ft have nearly identical computational costs because they share the same Faster R-CNN architecture, while FUSED-Net introduces only a lightweight feature fusion module. In contrast, A-RPN incurs substantially higher inference time due to support-set processing during inference, while DeFRCN requires considerably more GPU memory and inference time. CD-FSOD has the lowest computational cost; nevertheless, FUSED-Net achieves approximately 62\% higher performance without additional computational overhead over the standard Faster R-CNN framework. These results demonstrate that FUSED-Net provides a favorable balance between detection performance and computational efficiency.

Table 3: Computational cost analysis among SOTA methods and FUSED-Net. IT denotes average inference time (in seconds) for a sample, and PGM denotes peak GPU memory (in GB).

Methods | GFLOPs | IT | PGM

----------|--------|-------|-----

TFA w/cos | 276.92 | 0.144 | 0.71

A-RPN | 283.89 | 1.059 | 3.26

DeFRCN | 186.22 | 0.568 | 10.44

FRCN-ft | 276.92 | 0.145 | 0.71

CD-FSOD | 197.22 | 0.069 | 0.80

FUSED-Net | 276.92 | 0.146 | 0.71

The corresponding discussion and table have been added to the Computational Cost Analysis subsection in Page 16 (Line 605 onwards).

Reviewer 1, Concern 4:

We thank the reviewer for suggesting these recent studies. Following the recommendation, we have incorporated the three suggested works into the second paragraph of the Introduction section to provide a broader discussion of recent advances in object detection, including geometry-guided point generation, structure-aware transformer architectures, and multi-granularity prompt learning. These studies help contextualize our work within the recent development of object detection techniques, while our work specifically focuses on few-shot traffic sign detection under limited labeled data.

Reviewer 2, Concern 1:

We thank the reviewer for this comment. We would like to clarify that, in response to the related concern raised by R\#1C\#2, we have added a detailed augmentation ablation study in the revised manuscript. Specifically, we compare Color Jitter, random cropping, and horizontal flipping, both individually and in combination, across the 1-shot, 3-shot, 5-shot, and 10-shot settings on the BDTSD dataset. The corresponding results are reported in Table 1, with the detailed analysis provided in the Ablation Study in Page 18 (Line 671 onwards).

Table 1: (Repeated for Convenience) Comparison of mAP Performance of FUSED-Net with different augmentation techniques on the Bangladeshi Traffic Sign Detection (BDTSD) Dataset under 1-shot, 3-shot, 5-shot, and 10-shot scenarios.

Color Jitter | Crop | Horizontal Flip | 1-shot | 3-shot | 5-shot | 10-shot | Mean

-------------|------|-----------------|--------|--------|--------|---------|------

✓ | ✗ | ✗ | 28.22 | 44.49 | 47.23 | 54.95 | 43.72

✗ | ✓ | ✗ | 25.00 | 41.42 | 46.35 | 54.46 | 41.81

✗ | ✗ | ✓ | 23.03 | 36.02 | 42.92 | 54.97 | 39.23

✓ | ✓ | ✗ | 26.53 | 42.42 | 46.70 | 51.18 | 41.71

✓ | ✗ | ✓ | 21.53 | 35.75 | 42.81 | 52.87 | 38.29

✗ | ✓ | ✓ | 21.27 | 36.85 | 42.00 | 55.37 | 38.87

✓ | ✓ | ✓ | 23.42 | 40.05 | 44.78 | 50.43 | 39.67

As shown in the table, Color Jitter alone achieves the highest mean mAP (43.72) among all evaluated configurations, providing empirical support for our choice of augmentation strategy. We have also clarified the implementation details of Color Jitter in the Implementation Details section in Page 12 (line 463 onwards).

Thus, the requested comparative experimental details and quantitative justification have been incorporated into the revised manuscript.

Reviewer 2, Concern 2:

We thank the reviewer for emphasizing the importance of clearly reporting the training hyperparameters to improve the reproducibility of our work. To address this concern, we have added a summary of the key optimization and training settings used during both the base pre-training stage and the few-shot fine-tuning stage, as presented in Table 4. Unless otherwise specified, the implementation follows the standard Detectron2 Faster R-CNN training protocol with a ResNet-101-FPN backbone.

Table 4: Key training hyperparameters used during base pre-training and few-shot fine-tuning.

| Hyperparameter | Base Pre-training | Few-shot Fine-tuning |

| --------------------- | ----------------- | ---------------------- |

| Initial learning rate | 0.0025 | 0.0025 |

| Images per batch | 2 | 2 |

| Optimizer | SGD | SGD |

| Momentum | 0.9 | 0.9 |

| Weight Decay | 1 × 10−⁴ | 1 × 10−⁴ |

| Maximum iterations | 90000 | 1500, 2500, 2500, 3000 |

| Learning rate decay | 60000 | 1000, 2000, 2000, 2500 |

| Dataset | MTSD | BDTSD |

| Number of classes | 272 | 15 |

Both base training and fine-tuning are performed on a single GPU with limited memory, thus the batch size is reduced to two images, and the learning rate is scaled linearly to 0.0025 following the standard linear learning-rate scaling rule (https://github.com/facebookresearch/detectron2/issues/1128). The model is trained for 90,000 iterations, with the learning rate decayed at 60,000 iterations for base training. During few-shot fine-tuning, the backbone is initialized from the pre-trained Faster R-CNN ResNet-101-FPN model. The fine-tuning stage is performed for 1,500 iterations in the 1-shot setting, with the number of training iterations increasing proportionally for higher-shot settings. The learning rate is decayed at an appropriate stage during training for each setting. The corresponding training schedules and learning rate decay points for all shot configurations are reported accordingly.

Furthermore, we have also incorporated the analysis and Table 4 into the Implementation Details of the manuscript in Page 12 (Line 455 onwards), to address this concern.

Reviewer 2, Concern 3:

We sincerely thank the reviewer for pointing this out. We have retained the original resolution of the dataset images used in the manuscript without additional downsampling or quality degradation, while the majority of our figures are provided in PDF format to preserve high resolution. We have also revised the caption of Figure 7 (Figure 8 of the original manuscript) to provide a more detailed explanation of the observed failure mode, clarifying that the model successfully localizes the extremely distant traffic sign but incorrectly classifies it due to its small visual footprint and complex symbol. Moreover, the caption of Figure 5 (Figure 6 of the original manuscript) has been updated to clearly indicate that the ± values shown on the right side of each bar represent the quantitative variations corresponding to the 95\% confidence intervals obtained from the statistical significance analysis.

Reviewer 2, Concern 4:

We thank the reviewer for this insightful question regarding the role of the scaling factor (γ) in the cosine similarity-based classifier.

The scaling factor (γ) is adopted following the Frustratingly Simple Few-Shot Object Detection (TFA w/cos) framework [2], which employs a fixed scaling factor of 20. In their empirical study, the authors demonstrate that combining instance-level feature normalization with a cosine similarity-based classifier effectively reduces intra-class variance and improves the detection performance of novel classes, particularly in low-data few-shot scenarios, while maintaining the performance of base classes. Since FUSED-Net follows the same cosine similarity-based classification framework, we adopt the same empirically validated scaling factor to ensure a fair comparison with the baseline and to leverage its demonstrated effectiveness.

From a methodological perspective, embedding normalization projects both feature embeddings and classifier weights onto the unit hypersphere, causing classification to depend solely on the angular similarity between them. Consequently, the cosine similarity values are bounded within the range ([-1,1]), resulting in logits with relatively small magnitudes. The scaling factor (γ) rescales these logits before the softmax operation, producing a sharper probability distribution and stronger optimization gradients. This encourages feature embeddings from the same class to form more compact clusters while increasing the angular separation between different classes, thereby reducing intra-class variance and improving class discriminability.

This behavior is particularly beneficial for Bangladeshi few-shot traffic sign detection. Images of the same traffic sign often exhibit substantial appearance variations due to illumination changes, viewing angles, weather conditions, motion blur, partial occlusion, sign deterioration, and background clutter. At the same time, only a handful of training examples are available for each novel category. Under these conditions, embedding normalization together with the scaling factor of 20 encourages feature embeddings belonging to the same traffic sign category to remain tightly clustered while maintaining greater angular separation from visually similar categories. As a result, the classifier becomes more robust to the large intra-class variations commonly observed in Bangladeshi traffic signs and generalizes more effectively in the few-shot setting.

To address this concern, we have expanded the Implementation Details section in Page 13 of the updated manuscript (Line 478 onwards) to provide a detailed discussion of the rationale for selecting γ=20, its effect on feature normalization and class separation, and its relevance to the intra-class variations observed in Bangladeshi traffic signs.

Reviewer 2, Concern 5:

We thank the reviewer for suggesting these studies. The suggested references and corresponding discussion have been added to the first paragraph of the Introduction, following the discussion of the challenges associated with reliable traffic sign perception in real-world autonomous driving environments. Specifically, we have expanded the discussion of the challenges associated with reliable traffic-sign perception in autonomous driving by highlighting recent research on meta-adversarial defense, robust pre-training and fine-tuning for traffic sign classification, and self-adaptive defense against attacks on vision-language models. This addition provides a broader context regarding the robustness and reliability requirements of perception systems in safety-critical autonomous driving applications, while our work focuses specifically on addressing the complementary challenge of few-shot traffic sign detection under limited labeled data.

We believe these latest revisions have resulted in a much stronger and more complete manuscript. We thank you again for your time and consideration.

References

[1] Chen T, Kornblith S, Norouzi M, Hinton G. A Simple Framework for Contrastive Learning of Visual Representations. In: III HD, Singh A, editors. Proceedings of the 37th International Conference on

Machine Learning. vol. 119 of Proceedings of Machine Learning Research. PMLR; 2020. p. 1597-607. Available from: https://proceedings.mlr.press/v119/chen20j.html.

[2] Wang X, Huang T, Gonzalez J, Darrell T, Yu F. Frustratingly Simple Few-Shot Object Detection. In: III HD, Singh A, editors. 37th International Conference on Machine Learning (ICML). vol. 119

of Proceedings of Machine Learning Research. PMLR; 2020. p. 9919-28. Available from: https://proceedings.mlr.press/v119/wang20j.html. doi:10.5555/3524938.3525858.

Attachment Submitted filename: r1-response-july2026.pdf

# Peer review: Decision Letter 1

**DOI:** 10.1371/journal.pone.0359293.r003

**Authors:** Baicang Guo

13 Sep 2026

FUSED-Net: Detecting traffic signs with limited data

PONE-D-26-29998R1

Dear Dr. Kabir,

We’re pleased to inform you that your manuscript has been judged scientifically suitable for publication and will be formally accepted for publication once it meets all outstanding technical requirements.

Within one week, you’ll receive an e-mail detailing the required amendments. When these have been addressed, you’ll receive a formal acceptance letter and your manuscript will be scheduled for publication.

An invoice will be generated when your article is formally accepted. Please note, if your institution has a publishing partnership with PLOS and your article meets the relevant criteria, all or part of your publication costs will be covered. Please make sure your user information is up-to-date by logging into Editorial Manager at Editorial Manager® and clicking the ‘Update My Information' link at the top of the page. For questions related to billing, please contact billing support.

If your institution or institutions have a press office, please notify them about your upcoming paper to help maximize its impact. If they’ll be preparing press materials, please inform our press team as soon as possible -- no later than 48 hours after receiving the formal acceptance. Your manuscript will remain under strict press embargo until 2 pm Eastern Time on the date of publication. For more information, please contact onepress@plos.org.

Kind regards,

Baicang Guo

Academic Editor

PLOS One

Additional Editor Comments (optional):

Reviewers' comments:

Reviewer's Responses to Questions

**Comments to the Author**

Reviewer #1: All comments have been addressed

Reviewer #2: All comments have been addressed

**********

2. Is the manuscript technically sound, and do the data support the conclusions?

Reviewer #1: Yes

Reviewer #2: Yes

**********

3. Has the statistical analysis been performed appropriately and rigorously?

Reviewer #1: Yes

Reviewer #2: Yes

**********

4. Have the authors made all data underlying the findings in their manuscript fully available?

The PLOS Data policy

Reviewer #1: Yes

Reviewer #2: Yes

**********

5. Is the manuscript presented in an intelligible fashion and written in standard English?

Reviewer #1: Yes

Reviewer #2: Yes

**********

Reviewer #1: The revised manuscript has addressed all comments, and the manuscript is now suitable for acceptance.

Reviewer #2: The authors make a good revison. All my concerns have been appropriately solved and I think it can be accepted now.

**********

what does this mean?). If published, this will include your full peer review and any attached files.

If you choose “no”, your identity will remain anonymous but your review may still be made public.

**Do you want your identity to be public for this peer review?**  For information about this choice, including consent withdrawal, please see our Privacy Policy

Reviewer #1: No

Reviewer #2: No

**********

# Editor report: Acceptance letter

**DOI:** 10.1371/journal.pone.0359293.r004

**Authors:** Baicang Guo

PONE-D-26-29998R1

PLOS One

Dear Dr. Kabir,

I'm pleased to inform you that your manuscript has been deemed suitable for publication in PLOS One. Congratulations! Your manuscript is now being handed over to our production team.

At this stage, our production department will prepare your paper for publication. This includes ensuring the following:

* All references, tables, and figures are properly cited

* All relevant supporting information is included in the manuscript submission,

* There are no issues that prevent the paper from being properly typeset

You will receive further instructions from the production team, including instructions on how to review your proof when it is ready. Please keep in mind that we are working through a large volume of accepted articles, so please give us a few days to review your paper and let you know the next and final steps.

Lastly, if your institution or institutions have a press office, please let them know about your upcoming paper now to help maximize its impact. If they'll be preparing press materials, please inform our press team within the next 48 hours. Your manuscript will remain under strict press embargo until 2 pm Eastern Time on the date of publication. For more information, please contact onepress@plos.org.

You will receive an invoice from PLOS for your publication fee after your manuscript has reached the completed accept phase. If you receive an email requesting payment before acceptance or for any other service, this may be a phishing scheme. Learn how to identify phishing emails and protect your accounts at https://explore.plos.org/phishing.

If we can help with anything else, please email us at customercare@plos.org.

Thank you for submitting your work to PLOS One and supporting open access.

Kind regards,

PLOS One Editorial Office Staff

on behalf of

Professor Baicang Guo

Academic Editor

PLOS One
